# Quality of life of persons with young-onset dementia: Repeated self-reported assessment over two years from diagnosis

**DOI:** 10.1177/13872877251371305

**Published:** 2025-08-29

**Authors:** Malin Aspö, Leonie NC Visser, Berit Seiger Cronfalk, Miia Kivipelto, Anne-Marie Boström

**Affiliations:** 1 Department of Neurobiology, Care Sciences and Society, Division of Clinical Geriatrics, Karolinska Institutet, Stockholm, Sweden; 2 Theme Inflammation and Aging, Karolinska University Hospital, Stockholm, Sweden; 3 Department of Medical Psychology, Amsterdam University Medical Center, University of Amsterdam, Amsterdam Public Health, Amsterdam, The Netherlands; 4 Department of Neurobiology, Care Sciences and Society, Division of Nursing, Karolinska Institutet, Stockholm, Sweden; 5 Institute of Public Health and Clinical Nutrition, University of Eastern Finland, Kuopio, Finland; 6 Department of Research and Development, Stockholms Sjukhem Foundation, Stockholm, Sweden

**Keywords:** Alzheimer's disease, dementia, health-related quality of life, longitudinal, QoL-AD, quality of life, RAND-36, young-onset dementia

## Abstract

**Background:**

To provide adequate support for persons with young-onset dementia (YOD) at the optimal time point, we need to know how their quality of life (QoL) changes over time.

**Objective:**

The aim was to describe trajectories in self-reported QoL and health related QoL (HRQoL) scores over a period of two years in persons recently diagnosed with YOD, and to explore the feasibility of repeated longitudinal QoL assessments.

**Methods:**

The study has a longitudinal explorative design. Persons with YOD (n = 33) were recruited at time of diagnosis and asked to complete a questionnaire including QoL-AD and RAND-36, every six months for two years. Questionnaire data were analyzed using Wilcoxon signed-rank tests. Differences between “completers” and “non-completers” were assessed using the independent-samples Mann-Whitney U tests or Pearson's Chi-square tests. Trajectories were visually explored by plotting individual and group scores over time.

**Results:**

Over time, we found significant differences in the RAND-36 domain “energy/fatigue”, with higher scores reported at 12 months (p = 0.047) and 24 months (p = 0.026) compared to shortly after diagnosis. Scatterplots of individual trajectories showed great variation in scores, without any clear patterns. The difference at baseline between the “non-completers” and the “completers” was that more participants in the “completers” group also participated in a yearly interview study.

**Conclusions:**

The study highlights that even if QoL or HRQoL seems to be stable over time at group level, individual trajectories may show great variation. Research is needed to evaluate sensitive and clinically relevant QoL instruments for persons with YOD.

## Introduction

Dementia is a syndrome resulting from underlying diseases such as Alzheimer's disease (AD), frontotemporal degeneration (FTD), or Lewy body disease (LBD). Dementia has a substantial impact on the everyday lives and the quality of life (QoL) of the persons diagnosed with dementia, as well as the lives of their loved ones.^1–4^ Persons diagnosed with dementia before the age of 65 years, i.e., those with young-onset dementia (YOD), have to deal with specific challenges, for example related to their work life and family roles. Their needs are not the same as those of older persons with dementia^5,6^ and, depending on the challenges they encounter as the disease progresses, the impact on their QoL might also be different.^7^ As we are not yet able to cure dementia, healthcare and research efforts need to focus on enhancing the well-being and QoL of persons living with dementia.^8^

There are several definitions of QoL and health-related quality of life (HRQoL). The World Health Organization (WHO) defines QoL as an: “*individual's perceptions of their position in life in the context of the cultural values and systems in which the live an in relation to their goals, expectations, standards and concerns” (page 4).*^9^ While QoL is related to the subjective experiences of life in general, HRQoL goes beyond this and includes the impact of the person's health status. Hays and Morales^10^ define HRQoL as: “*how health impacts on an individual's ability to function and his or her perceived well-being in physical, mental and social domains of life” (page 350).* The Quality of Life-Alzheimer's Disease^11^ (QoL-AD) scale is frequently used to measure QoL in dementia,^12^ and the generic RAND-36^13^ and SF-36^14^ are often used to assess HRQoL in different settings.^15–20^

However, there are some challenges in measuring QoL and HRQoL in people with dementia, such as high drop-out rates in longitudinal studies.^21,22^ In addition, a person requires a certain level of insight into their own deficits and an awareness of the disease when reporting their QoL. It has previously been shown that persons with low awareness/insight, which is a symptom of dementia, tend to rate their QoL higher.^23^ For this reason, QoL instruments often rely on proxy report, for example from the informal caregiver or nursing staff, and several do not include reporting by the persons with dementia themselves.^12,20^ Previous research has shown differences between self-reported QoL and proxy-reported QoL.^20,23–27^ Self-reported QoL tends to remain stable over time as the disease progresses, while the proxy report shows decline.^23–27^ One study also found this discrepancy in younger persons with dementia.^28^ However, there are several factors that may influence the proxy report, such as (the increasing) caregiver burden related to cognitive and functional decline of the person with dementia.^23,29,30^ Moreover, a proportion of people with YOD live alone or spend a lot of time alone, for example because their life partner is still working, and might therefore not have anyone who can provide a reliable proxy report. Even though it is argued in the literature that persons with dementia might overrate their own QoL, it is still important to take the person's own perspective into account, and not solely rely on proxy ratings, when attempting to understand the QoL of persons living with dementia.^24,29^

From a healthcare perspective, we specifically need insight into how QoL changes over time and if time points can be identified when the individual with YOD reports significantly higher or lower levels of QoL. With such knowledge, adequate support and care could be provided at an optimal point in time. The possibility to measure changes over time is one of the main advantages of a longitudinal study design.^31^ However, there are some challenges when it comes to repeated self-report measurements, especially in people with neurodegenerative diseases, including the risk of incomplete follow-up.^31^ Longitudinal dementia research, therefore, often faces relatively high dropout rates, and those who complete the study might differ from the dropouts,^32^ for example in that those who drop out may have more severe cognitive decline or lower QoL at the start. The aim of this longitudinal study is, therefore, twofold. First, we aim to describe trajectories in self-reported QoL and HRQoL scores over two years in persons recently diagnosed with YOD. Second, to explore the feasibility of repeated longitudinal QoL assessments, including any differences between participants who complete the two-year data collection and those who drop-out prior to study completion.

## Methods

### Study design

This longitudinal study is part of a larger, five-year project collecting self-reported quantitative data concerning QoL and HRQoL and, in a subsample, qualitative data from yearly interviews. The participants were included shortly after being diagnosed with YOD and then followed for five years. In this paper we focus on the initial period following diagnosis and will report on the questionnaire data collected during the first two years of the project.

#### Setting and participants

Participants were consecutively recruited between May 2018 and May 2021 from two specialized memory clinics in an urban area of Sweden. These clinics serve persons with cognitive complaints and suspected dementia, of which a substantial proportion are relatively young. Prior to being referred to these clinics, the patients underwent basic assessments, such as blood sampling, computed tomography (CT), and Mini-Mental State Examination (MMSE). The diagnostic work-up at the clinic is conducted by a multidisciplinary team, and includes medical and neurological examinations, cognitive testing by neuropsychologists, and blood tests, as well as lumbar puncture and magnetic resonance imaging (MRI) for AD-biomarker analysis. In the clinic, approximately 25% of the patients are diagnosed with dementia, 25% with mild cognitive impairment (MCI), and 50% with subjective cognitive decline (SCD).^33^

Patients were considered eligible for participation if they fulfilled the following criteria: age below 65 years; recently diagnosed with dementia; scores ≥ 24 on the MMSE or ≥21 on the Montreal Cognitive Assessment (MoCa); no previously diagnosed conditions of impaired cognition; able to communicate and having the capacity to consent to participation. Patients who fulfilled the inclusion criteria were asked by a physician if they would be interested in participating in the study. Those who expressed interest were contacted by the first author. A total of 49 patients were considered eligible and asked by the physician. Of these, 16 were not included, seven did not fulfill the inclusion criteria, one declined participation after receiving detailed information about the study, four could not be reached, and four were not contacted within a reasonable time after diagnosis due to logistical reasons related to the COVID-19 pandemic. Based on clinical data approximately 200 patients were diagnosed with YOD during the time of recruitment. Out of these, 70 had a MMSE score of ≥24 or a MoCa score of ≥21. Unfortunately, there is no information regarding if these patients fulfilled the remaining inclusion criteria and why they were not informed about the study by the physicians. In total, 33 participants were included in the study, all within two months of receiving their diagnosis. A subsample of 15 participants were consecutively asked if willing to participate in qualitative interviews in addition to responding to the questionnaires.

#### Data collection and instruments

Data were collected at five time points: shortly after diagnosis (T1), and then after six (T2), 12 (T3), 18 (T4), and 24 (T5) months. At each time point, participants were asked to complete a questionnaire consisting of two validated instruments for measuring QoL and HRQoL: Quality of Life—Alzheimer's Disease (QoL-AD)^11^ and RAND-36.^13^ Participants were provided with the first questionnaire (T1) during a personal meeting with the first author. The four subsequent questionnaires were either sent to their home address by post for self-report or answered in a phone call with the first author, based on the participant's preferences.

QoL-AD is a disease-specific instrument frequently used for measuring QoL in dementia.^12^ The instrument consists of 13 items: *Physical health, Energy, Mood, Living situation, Memory, Family, Marriage, Friends, Self as a whole, Ability to do chores around the house, Ability to do things for fun, Money,* and *Life as a whole*, which respondents answer on a 4-point scale: poor, fair, good, or excellent.^11,34^ The instrument is valid, reliable, and sensitive to change, and can be completed by persons with mild to moderate dementia.^34,35^ QoL-AD has both a patient and a proxy report^11^; however, the instrument has been used in previous studies and judged to be valid and reliable, even without the proxy report.^35,36^ In this study, we have only included the self-reported part of the instrument. A self-report total score is normally calculated, which can range from 13 to 52. In the present study, Cronbach's alpha coefficient was calculated for baseline data to assess internal consistency and reliability of the instrument, showing a coefficient value of 0.87, which is considered satisfactory.^37^

RAND-36 is one of the most frequently used generic instruments for measuring HRQoL.^10^ It consists of 35 items covering 8 dimensions: *Physical functioning (10 items), Role limitations caused by physical health problems (4 items), Pain (2 items), General health perceptions (5 items), Energy/fatigue (4 items), Social functioning (2 items), Role limitations caused by emotional problems (3 items), and Emotional wellbeing (5 items),* and one item assessing change in perceived health during the past year.^10,13^ Three of the domains (“Physical functioning”, “Role limitations caused by physical health problems”, and “Pain”) focus on physical aspects, three domains (“Emotional wellbeing”, “Social functioning”, and “Role limitations caused by emotional problems”) focus on mental aspects, and two domains (“General health perceptions” and “Energy/fatigue”) cover both physical and mental aspects of HRQoL.^10^ The instrument is valid and reliable for use in longitudinal studies.^10^ The instrument was developed for self-administration but can also be administered by an interviewer by phone or face-to-face.^10^ The RAND-36 is a license free version of the commercial SF-36. Previous studies conclude that SF-36 seems to be suitable for persons with mild to moderate dementia^38^ who have insight into their deficits and a MMSE score greater than 16.^20^ For each of the domains, scores can range from 0 to 100, a higher score indicating better HRQoL.^10^ Cronbach's alpha coefficients were calculated for each of the domains of RAND-36. At baseline, all domains showed acceptable^37^ alpha values (above 0.70): “Physical functioning” (0.83), “Role limitations caused by physical health problems” (0.82), “Pain” (0.83), “General health perceptions” (0.75), “Energy/fatigue” (0.86), “Social functioning” (0.75), “Role limitations caused by emotional problems” (0.73), “Emotional wellbeing” (0.85).

### Analysis

Statistical analyses were performed in SPSS version 28.0.1.1 (14).

#### Grouping into completers and non-completers

The sample was divided into two groups to explore differences between participants who completed the questionnaire at each time point and those who did not. A participant was considered a “completer” if he/she had responded to all the questionnaires, that is completed the questionnaire at all five time points; 20 participants were deemed completers. The 13 participants who were considered “non-completers” were those who had either dropped out at some point during data collection (n = 6) or had missed one or more time point measurements (n = 7). Drop-outs were those who either withdrew their consent (n = 4) or were excluded due to not returning the questionnaire at three consecutive time points (n = 2). Seven participants in the “non-completer” group did not return the questionnaire at one (n = 4) or two (n = 3) time points. At month 18 (T4), the questionnaire was mistakenly not sent to two of the participants; these were included in the non-completers group.

#### Handling of missing data on QoL or HRQoL instruments

There were missing data for both the QoL-AD total scores and the RAND-36 domain scores among the “completers” and the “non-completers”. Most commonly, the total score for QoL-AD was missing because we did not impute scores if the participant had omitted one or more items. For the QoL-AD questionnaire, the total score was calculated by summing up item scores^11^ and if one or more items were missing, then the total score was not calculated. Domain scores for RAND-36 were calculated using the excel file provided from Registercentrum Sydost (www.rcso.se), where a domain score is calculated if at least 50% of the items within that domain were responded to.

#### Qol and HRQoL scores over time

Descriptive statistics were calculated for QoL-AD total scores and for the eight domains of RAND-36. Changes in QoL and HRQoL scores over time were then examined. We compared QoL-AD total scores and scores for each of the domains of the RAND-36 at each time point to the score at T1 (immediately after diagnosis). Due to skewness and data not being normally distributed, non-parametric testing was conducted using the related-samples Wilcoxon signed-rank test, with a significance level set at 0.05. The single item on RAND-36, assessing change in perceived health during the last year, was analyzed using Pearson's Chi-2 test.

In addition, scatter plots were constructed to visually explore patterns at both group level and individual level. Scatter plots were first constructed for the QoL-total score and the eight RAND-36 domain scores displaying group medians across all time points. To inspect trajectories of each individual participant, scatter plots for the QoL-AD total score and for each domain of the RAND-36 were constructed. These scatter plots displayed the individual scores for each of the participants on the specific domain over time. In addition, we also created separate scatter plots for the completers and non-completers.

#### Baseline differences between completers and non-completers

We explored initial differences between completers and non-completers using data from the baseline questionnaire. These data included sex, age, marital status, educational level, occupation, and if the participant had children (including children of any age, children younger than 18 years, and children living at home). We also retrieved data from patients’ medical records regarding cognitive screening tests (the MMSE and MoCa scores) and diagnosis. A final variable concerned whether the participant was part of the qualitative sub-study in the larger longitudinal project in which a subsample was interviewed yearly by the researcher (main author).

An independent-samples Mann-Whitney U test was performed to assess demographic differences between the groups regarding numerical variables (age, MMSE and MoCa scores). For the remaining variables, i.e., the categorical variables, differences between the completers and non-completers were assessed using Pearson's Chi-2 test. Differences in QoL and HRQoL scores could only be tested at T1, that is immediately after diagnosis, because of low availability of data in the non-completer group at follow up measurements. To assess differences in QoL-AD total score and RAND-36 domain scores at baseline, non-parametric testing was conducted using the independent-samples Wilcoxon signed-rank test, with a significance level set at 0.05.

## Results

### Description of sample

In total, 33 participants with an average age of 60.0 (SD = 4.0) years were included in the study (Table 1). The most common diagnosis was dementia due to Alzheimer's disease (51.5%). Average MMSE scores were 27.0 (SD = 1.6), with individual scores ranging from 24 to 30, and average MoCa scores were 22.5 (SD = 3.0), ranging from 15 to 28. The majority of participants were female (69.7%), married (57.6%), and had a university degree or higher educational level (68.8%). All except two participants had children (93.9%), and four participants had children under 18 years (12.1%). Approximately half of the participants were either on sick leave (41.4%) or had retired early (17.2%) at the time of diagnosis and completion of the first questionnaire.

**Table 1.** Baseline characteristics.

	All participants	Completers	Non-completers	p^a^
	N = 33	N = 20	N = 13	
**Female N (%)**	23 (69.7)	14 (70.0)	9 (69.2)	0.963
**Age**	N = 33	n = 20	n = 13	0.413
Mean (SD)	60.0 (±4.0)	60.4 (±3.9)	59.3 (±4.2)	
Median	61.0	61.0	61.0	
Range	49–65	49–65	51–64	
**Diagnosis N**	N = 33	N = 20	N = 13	0.578
Dementia due to Alzheimer's disease	17 (51.5)	9 (45.0)	8 (61.5)	
Prodromal Alzheimer's disease	11 (33.3)	8 (40.0)	3 (23.1)	
Other**^b^**	5 (15.2)	3 (15.0)	2 (15.4)	
**MMSE score**	N = 30	N = 20	N = 10	0.880
Mean (SD)	27.0 (±1.6)	27.1 (±1.4)	26.7 (±2.1)	
Median (range)	27.0 (24–30)	27.0 (24–30)	27.5 (24–29)	
**MoCA score**	N = 31	N = 18	N = 13	0.211
Mean (SD)	22.5 (±3.0)	23.2 (±2.6)	21.5 (±3.4)	
Median (range)	23.0 (15–28)	23.0 (19–21)	22.0 (15–26)	
**Marital status N**	N = 33	N = 20	N = 13	0.193
Married	19 (57.6)	14 (70.0)	5 (38.5)	
Living with a partner	3 (9.1)	1 (5.0)	2 (15.4)	
Living alone	10 (30.3)	4 (20.0)	6 (46.2)	
Widowed	1 (3.0)	1 (5.0)	0 (0.0)	
**Children N**	N = 33	N = 20	N = 13	
Children of any age (including adults)	31 (93.9)	19 (95.0)	12 (92.3)	0.751
Children under 18 years	4 (12.1)	3 (15.0)	1 (7.7)	0.530
Children living at home	6 (18.2)	4 (20.0)	2 (15.4)	0.737
**Education N (%)**	N = 32	N = 20	N = 12	0.107
Secondary education (lower and upper)	4 (12.5)	2 (10.0)	2 (16.7)	
Post-secondary education	6 (18.8)	6 (30.0)	0 (0.0)	
University	22 (68.8)	12 (60.0)	10 (83.3)	
**Occupation N (%)**	N = 29	N = 17	N = 12	0.426
Student	1 (3.4)	0 (0.0)	1 (8.3)	
Working full-time	6 (20.7)	3 (17.6)	3 (25.0)	
Working part-time	5 (17.2)	4 (23.5)	1 (8.3)	
On sick leave	12 (41.4)	6 (35.3)	6 (50.0)	
Retired	5 (17.2)	4 (23.5)	1 (8.3)	
**Included in interview study N (%)**	15 (45.5)	12 (60.0)	3 (23.1)	0.037*

a Comparing the completers and the non-completers.

b Atypical AD/PCA (n = 3); unspecified dementia (n = 1); Mixed dementia (n = 1).

*Significant difference (p-value <0.05).

There were no differences in characteristics between completers (n = 20) and non-completers (n = 13) in terms of sex, age, diagnosis, cognition, marital status, children, education, and occupation (see Table 1 for p-values, right hand column). One significant difference (p = 0.037) was found when comparing characteristics between the groups, which showed that completers were more likely to be enrolled in the interview sub-study. In total, 15 participants also took part in the interview study: 12 were in the completers group (12/20; 60.0%) and three (3/13; 23.1%) in the non-completers group.

### Self-reported QoL scores over time

Table 2 shows descriptive statistics for all participants with a QoL-AD total score at each time point. A QoL-AD total score was available for 21 to 25 participants, depending on the time point. Median scores varied from 38.0 to 41.0. Individual scores presented at each time point, varied enormously, for example ranging from 17 to 50 at T2. Completers and non-completers were compared at T1, showing no difference in QoL-AD total score at baseline.

**Table 2.** QoL-AD total score: descriptive statistics and Wilcoxon signed-rank test.

	Descriptive statistics					Wilcoxon signed-rank test^a^		
Time point	N^b^	Mean	SD	Median	min-max	N^c^	Z	p
**T1 (Diagnosis)**	25	39.5	±6.3	40.0	22–50	N/A	N/A	N/A
**T2 (Month 6)**	22	40.1	±7.5	41.0	17–50	17	−1.06^e^	0.287
**T3 (Month 12)**	25	40.6	±6.5	41.0	21–51	17	−0.03^e^	0.975
**T4 (Month 18)**	21	39.6	±6.1	38.0	30–50	15	−0.92^e^	0.359
**T5 (Month 24)**	21	40.1	±6.0	39.0	30–51	16	−0.09^d^	0.932

a Wilcoxon signed-rank test comparing each time point to T1 (Diagnosis).

b Number of participants with a valid score at this time point.

c Number of participants included in analysis. i.e., who had valid scores at T1 and at the time point being compared to T1.

d Based on positive ranks.

e Based on negative ranks.

Table 2 also shows results of the Wilcoxon signed-rank test analyses, comparing scores at each time point to those at T1, the measurement directly after diagnosis. Two total scores needed to be available to make these comparisons, which was the case for 15 to 17 participants, depending on the time point. No statistically significant differences were found when comparing follow-up scores to the score after diagnosis (T1).

### Self-reported HRQoL scores over time

Descriptive statistics of HRQoL scores are presented in Table 3. Across all time points and domains, we could calculate domain scores for 23 to 31 participants. We found no difference between completers and non-completers in any of the RAND-36 domain scores at baseline.

**Table 3.** RAND-36 domain scores: descriptive statistics and Wilcoxon signed-rank test.

	Descriptive statistics at each time point					Wilcoxon signed-rank test^a^		
Time point	N^b^	Mean	SD	Median	min-max	N^c^	Z	p
**Physical functioning**								
T1 (Diagnosis)	31	95.0	±11.0	100.0	55–100	N/A	N/A	N/A
T2 (Month 6)	29	92.9	±15.0	100.0	40–100	28	−1.31^d^	0.189
T3 (Month 12)	27	94.3	±13.1	100.0	40–100	25	−1.77^d^	0.077
T4 (Month 18)	25	89.8	±24.0	100.0	5–100	24	−1.56^d^	0.120
T5 (Month 24)	24	94.0	±12.2	100.0	50–100	22	−1.23^d^	0.219
**Role limitations caused by physical health problems**								
T1 (Diagnosis)	30	77.2	±33.1	100.0	0–100	N/A	N/A	N/A
T2 (Month 6)	29	85.3	±28.8	100.0	0–100	27	−0.99^e^	0.321
T3 (Month 12)	27	82.4	±31.6	100.0	0–100	24	−0.47^e^	0.640
T4 (Month 18)	25	81.7	±32.0	100.0	0–100	23	−0.26^d^	0.796
T5 (Month 24)	24	80.2	±34.6	100.0	0–100	22	−0.31^e^	0.754
**Pain**								
T1 (Diagnosis)	31	85.0	±22.5	100.0	20–100	N/A	N/A	N/A
T2 (Month 6)	29	82.9	±29.3	100.0	13–100	28	−0.69^d^	0.488
T3 (Month 12)	27	88.9	±19.6	100.0	45–100	25	−0.28^e^	0.777
T4 (Month 18)	24	88.3	±20.3	100.0	33–100	23	−0.09^e^	0.929
T5 (Month 24)	24	91.1	±20.7	100.0	23–100	22	−1.13^e^	0.258
**Social functioning**								
T1 (Diagnosis)	31	78.0	±25.5	88.0	13–100	N/A	N/A	N/A
T2 (Month 6)	29	79.5	±20.8	75.0	38–100	28	−0.36^e^	0.716
T3 (Month 12)	27	82.6	±21.7	88.0	25–100	25	−0.44^e^	0.661
T4 (Month 18)	25	73.6	±23.1	75.0	25–100	24	−1.24^d^	0.215
T5 (Month 24)	23	87.1	±22.1	100.0	25–100	21	−1.07^e^	0.284
**Role limitations caused by emotional problems**								
T1 (Diagnosis)	28	69.1	±37.4	83.5	0–100	N/A	N/A	N/A
T2 (Month 6)	29	75.9	±36.6	100.0	0–100	25	−0.25^e^	0.803
T3 (Month 12)	27	76.6	±34.7	100.0	0–100	23	−0.81^e^	0.416
T4 (Month 18)	25	72.6	±40.0	100.0	0–100	22	−0.06^d^	0.950
T5 (Month 24)	24	80.5	±34.0	100.0	0–100	21	−0.54^e^	0.589
**Emotional well-being**								
T1 (Diagnosis)	31	68.2	±17.3	72.0	32–96	N/A	N/A	N/A
T2 (Month 6)	29	68.4	±17.3	72.0	32–96	28	−0.88^e^	0.380
T3 (Month 12)	27	71.3	±15.3	72.0	40–96	25	−0.92^e^	0.360
T4 (Month 18)	25	70.6	±15.4	72.0	32–96	24	−0.61^e^	0.540
T5 (Month 24)	24	71.7	±15.6	72.0	40–92	22	−1.51^e^	0.132
**General health perceptions**								
T1 (Diagnosis)	31	67.7	±20.0	75.0	25–95	N/A	N/A	N/A
T2 (Month 6)	29	70.2	±17.5	70.0	30–100	28	−0.50^e^	0.614
T3 (Month 12)	27	68.0	±18.0	70.0	15–100	25	−0.67^d^	0.504
T4 (Month 18)	25	70.4	±21.5	75.0	30–100	24	−0.11^e^	0.909
T5 (Month 24)	24	71.0	±18.6	75.0	25–100	22	−0.51^e^	0.614
**Energy/fatigue**								
T1 (Diagnosis)	31	61.9	±20.0	65.0	0–90	N/A	N/A	N/A
T2 (Month 6)	29	64.3	±21.4	70.0	0–95	28	−1.52^e^	0.130
T3 (Month 12)	27	70.6	±16.3	70.0	35–95	25	−1.99^e^	0.047*
T4 (Month 18)	25	65.9	±21.6	65.0	15–100	24	−0.03^e^	0.976
T5 (Month 24)	24	71.5	±18.0	75.0	25–100	22	−2.22^e^	0.026*

a Wilcoxon signed-rank test comparing each time point to T1 (Diagnosis).

b Number of valid scores.

c Number of participants included in analysis. i.e., who had valid scores at T1 and at the time point being compared to T1.

d Based on positive ranks.

e Based on negative ranks.

*Sig. (2-sided test) p < 0.05.

Median scores for the three domains measuring physical aspects of HRQoL (“Physical functioning”, “Role limitations due to physical health”, and “Pain”) showed the highest scores and remained stable at 100.0 at each time point, although we observed a wide range of participants’ individual scores (see min-max).

For the three domains measuring mental aspects of HRQoL (“Social functioning”, and “Role limitations caused by emotional problems” and “Emotional wellbeing”) we observed more variation in median scores, with some lower scores compared to the domains focusing on physical aspects. Here again we observed a wide range of individual scores (see Min-Max).

The two domains measuring both physical and mental aspects of HRQoL (“General health perceptions” and “Energy/fatigue”) showed the lowest median scores. Median scores for “General health perceptions” varied between 70.0 and 75.0, and for “Energy/fatigue” between 65.0 and 75.0. There was a wide range again in the individual scores.

In addition, Table 3 displays the results of the Wilcoxon signed-rank tests. For each domain, we compared scores at T2, T3, T4 and T5 to the T1 score, resulting in 32 tests, based on data from 21 to 28 participants. Two of these showed significant differences: in the domain, “Energy/fatigue”, the scores at T3 (p = 0.047) and T5 (p = 0.026) were higher compared to T1, indicating an improvement in HRQoL.

In addition to the eight domains, RAND-36 also includes a single question assessing change in perceived health. At each time point, more than half of the sample rated their health to be about the same as one year previously (Table 4). Statistical analysis showed no significant differences between the different time points.

**Table 4.** RAND-36 change in perceived health during the last year.

	Much worse	Somewhat worse	About the same	Somewhat better	Much better
T1 (Diagnosis) N (%)	1 (3.2)	7 (22.6)	18 (58.1)	3 (9.7)	2 (6.5)
T2 (Month 6) N (%)	0 (0.0)	4 (14.3)	17 (60.7)	5 (17.9)	2 (7.1)
T3 (Month 12) N (%)	0 (0.0)	3 (11.1)	17 (63.0)	5 (18.5)	2 (7.4)
T4 (Month 18) N (%)	0 (0.0)	0 (0.0)	13 (52.0)	6 (24.0)	3 (12.0)
T5 (Month 24) N (%)	1 (4.2)	3 (12.5)	16 (66.7)	3 (12.5)	1 (4.2)

Pearson's Chi-2 test = 0.963.

### Visual exploration of data: individual trajectories

Since the statistical power to detect any differences was limited, we also explored the data visually. Scatter plots were constructed to visually inspect QoL and HRQoL scores over time, with separate lines for each participant. These plots show a great variation in scores, both between participants and for each participant over time. Participants started at different levels, some stayed relatively stable, others fluctuated scoring higher at a time point and then lower at the next, or vice versa.

**Figure 1.**
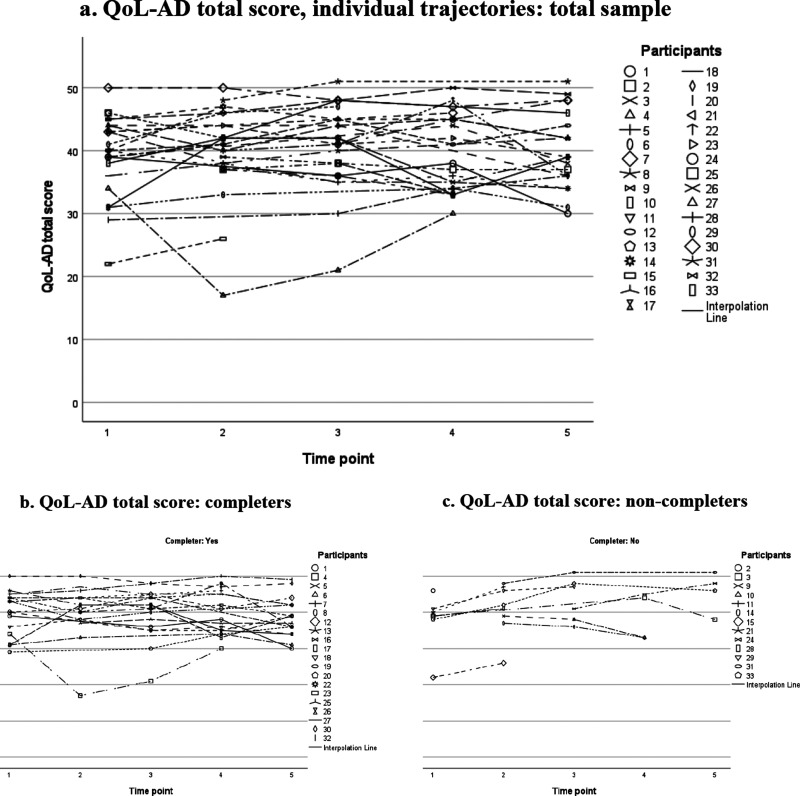
Quality of life (QoL-AD) scores over time: individual trajectories plotted for the total sample (a), and for completers (b) and non-completers (c) separately. Notes: Scores ranging from 13 to 52. Higher scores indicate better QoL.

When inspecting the individual trajectories for QoL-AD (Figure 1(a)), except for a few outliers all participants rated their QoL in the range of 30 to 50. Visually, we could detect no apparent patterns in QoL-AD total scores. This observation does not change if the sample is divided into completers and non-completers (see Figure 1(b, c)).

**Figure 2.**
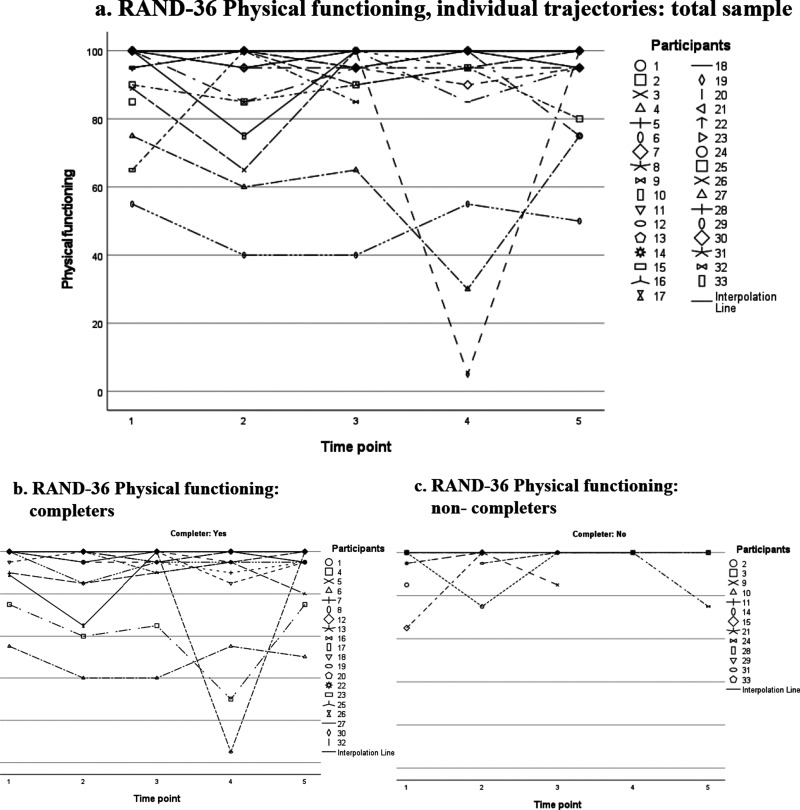
RAND-36 Physical functioning, domain scores over time: individual trajectories plotted for the total sample (a), and for completers (b) and non-completers (c) separately. Notes: Scores ranging from 0 to 100. Higher scores indicate better HRQoL.

Since the RAND-36 has eight domains, we have included a selection of scatter plots in the main text of this paper for illustrative purposes; the scatter plots for the other domains are available as Supplemental Material. Regarding the RAND-36 domain “Physical functioning” (Figure 2(a)), we observed that most participants rated their physical function as high at each time point, mostly within the range of 80 to 100, with only a few participants scoring lower at particular times. Dividing the group into completers (Figure 2(b)) and non-completers (Figure 2(c)) showed that lower scores are not specific to the non-completers group.

**Figure 3.**
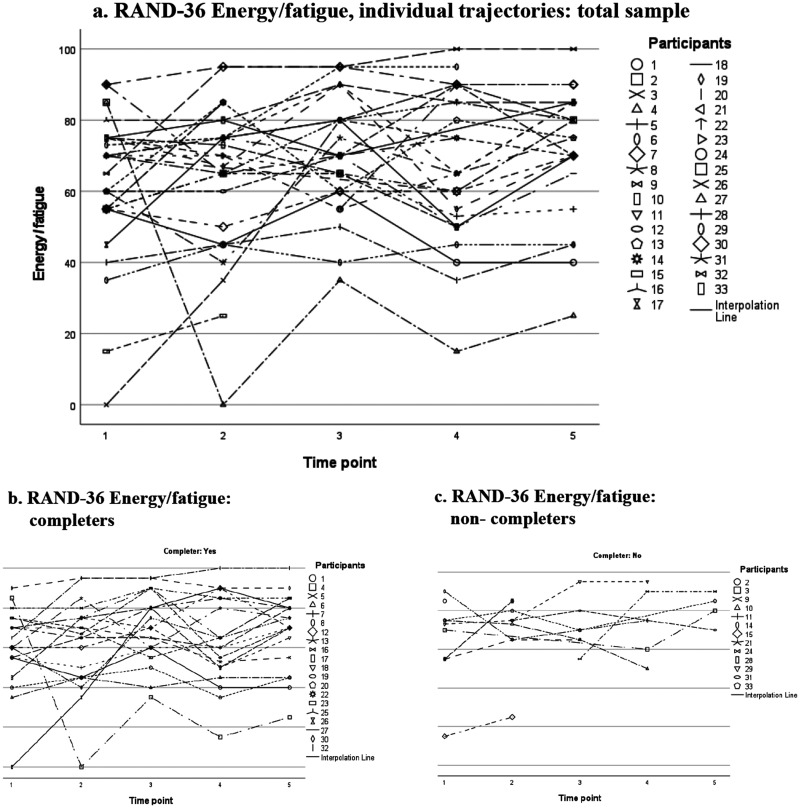
RAND-36 Energy/fatigue, domain scores over time: individual trajectories plotted for the total sample (a), and for completers (b) and non-completers (c) separately. Notes: Scores ranging from 0 to 100. Higher scores indicate better HRQoL.

Figure 3(a) displays scores on the domain “Energy/fatigue”, which was the only domain showing significant differences on the Wilcoxon signed-rank test. Nevertheless, we observed great variation in individual scores and individual trajectories, without any apparent patterns. This also holds true when inspecting scores for completers (Figure 3(b)) and non-completers (Figure 3(c)) separately.

**Figure 4.**
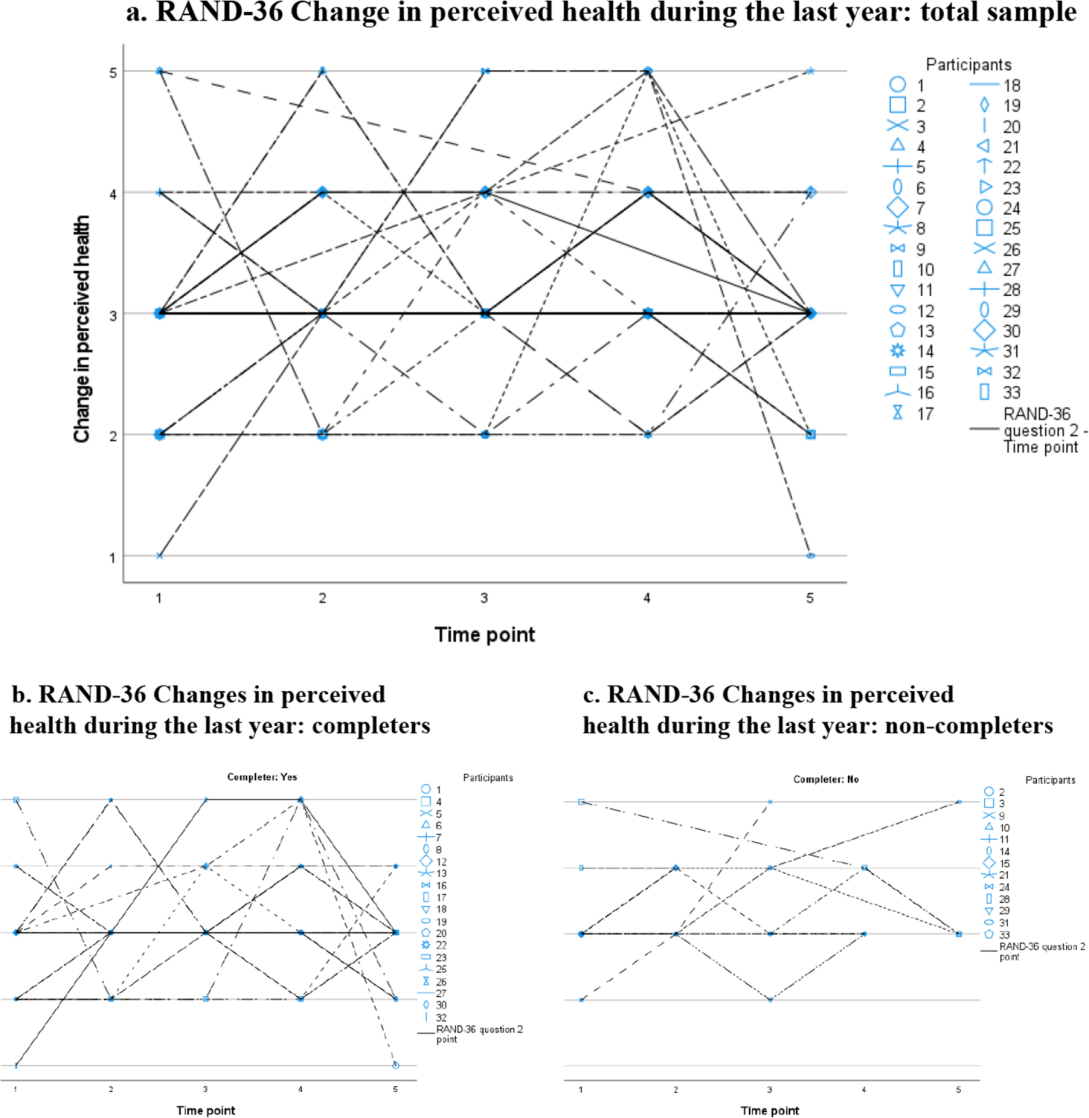
RAND-36 Changes in perceived health during the last year: individual trajectories plotted for the total sample (a), and for completers (b) and non-completers (c) separately. Notes: Changes in perceived health during the last year rated on a five-point Likert scale: 1. Much worse now than one year ago; 2. Somewhat worse now than one year ago; 3. About the same; 4. Somewhat better than one year ago; 5. Much better than one year ago.

At each time point, more than half of the participants reported no change in health during the last year. The scatter plots for this item also showed a great variation in individual scores and trajectories for the whole group (Figure 4(a)), as well as for the completers (Figure 4(b)) and non-completers (Figure 4(c)).

## Discussion

Our findings show that, at group level, QoL and HRQoL appear to be stable during the two years following diagnosis. However, individual scores showed a large variation over time, without any clear pattern or gradual decline. This variance was also seen for the RAND-36 domain “Energy/fatigue”, although statistically significant changes were seen at two time points compared to time of diagnosis. This highlights the importance of a person-centered approach, since only considering scores at group level, and not taking individual trajectories into account, might lead to an incorrect conclusion that QoL and HRQoL remain stable over time. The importance of looking beyond stable scores at group level is supported by Clare et al.^40^ who recently identified four distinct trajectories (stable, declining, improving, and stable with markedly lower QoL scores) in a large sample of older persons with mild to moderate dementia. As mentioned, we were not able to identify any distinct trajectories or patterns, which could partly be explained by our small sample size.

Previous studies on self-reported QoL-AD scores have mainly included participants of higher age and with lower MMSE scores,^11,24,35,41,42^ and have shown lower QoL scores compared to our sample. The self-reported QoL-AD scores in our study are closer to those presented in a sample of patients aged 50–90 years with prodromal or mild AD, with similar MMSE scores and disease duration as our sample.^36^ Lower QoL-AD scores have been shown to be associated with functional decline and reduced independence,^43^ which could explain why older persons with more cognitive impairment report lower QoL. Comparing the RAND-36 scores in our sample to the SF-36 scores in a study by Geschke et al.,^20^ our sample scored higher on the physical domains and reported similar or higher scores on the domains measuring mental aspects of HRQoL. In our sample, we observed a tendency towards a ceiling effect on several of the RAND-36 domains, especially those measuring physical aspects of HRQoL. This is not surprising, since the participants in our study are relatively young, with most having no co-morbidities or physical complaints. Compared to the general population (a sample with a mean age of 56.9 years, ranging from 20–100 years of age)^44^ our sample scored lower for the domains measuring mental aspects of HRQoL and the same or higher for physical domains. This emphasizes the clinical importance of focusing on the mental and social aspects of HRQoL, since these are more likely to be affected in persons with YOD.

We have only identified a few studies exploring self-reported QoL and HRQoL in younger persons with dementia, all cross-sectional.^45–48^ To our knowledge, our study is unique with its longitudinal approach and starting point at time of diagnosis. Assessing QoL and HRQoL can be challenging due to the nature of dementia, for example regarding participants’ memory complaints and lack of awareness. Many instruments for measuring QoL in dementia rely therefore partly or solely on proxy reports or are based on observations of the person's behavior.^12^ Hvidsten et al.^22^ collected longitudinal proxy-reported QoL data for younger persons in different phases of dementia. As in our study, they could not find any significant changes in QoL over a period of two years.

Based on our findings we could not identify a certain time point requiring extra attention during the first two years following diagnosis. However, we know from qualitative research that persons with YOD experience critical events and changes having a possible impact on QoL, such as receiving the diagnosis,^49,50^ withdrawal from working life,^49,51^ and facing an uncertain future.^49,52^ Additional critical points in dementia are related to the loss of driving license, behavioral symptoms, financial and healthcare-related considerations, and changes in care settings.^53,54^ Following persons with YOD with qualitative interviews for two years after being diagnosed, Johannessen et al.^52^ highlighted the existential threat of dementia, including changes in identity, as well as challenges in coping with the disease and social retraction. Since it is difficult to predict how fast dementia will progress for an individual,^55^ persons will encounter challenges related to dementia at different time points, with a variety of consequences affecting their QoL. Johannessen et al.^52^ further showed that persons with YOD, even those with severe short-term memory problems, were able to describe their experience of living with dementia. This indicates that using qualitative individual interviews may be a better approach to detect relevant changes over time, especially when considering the great variety of individual trajectories shown in our sample.

To our knowledge, there are no instruments addressing QoL and/or the specific needs of persons with YOD, especially those with mild dementia. The development of such a specific instrument would aid better understanding of the needs of persons with YOD, increasing the possibility to provide timely support. Based on our findings, we suggest that such an instrument should focus on the mental and social aspects of HRQoL, and cover areas relevant to the life situation of younger persons, including working life, children and meaningful activities,^49^ as well as existential aspects and signs of social retraction.^52^ In addition, traditional instruments measuring QoL and HRQoL often ask the respondent to recall information. For example, in RAND-36, the person is asked to consider the last month when responding to most questions.^13^ This can be especially challenging for someone experiencing cognitive impairments. Since memory complaints are a symptom of dementia, a more frequent momentary^56^ assessment of QoL may be preferred instead of asking the respondent to recall information.

### Methodological considerations

A strength of this study is that it included persons with YOD at time of diagnosis and has followed them for a period of two years. In addition, we included two measures: a measure of QoL and a measure of HRQoL. However, the study also has some limitations. Firstly, the sample size was small (n = 33), not normally distributed, and with several non-completers (41%). The low number of participants was mainly a result of the relatively small number of patients newly diagnosed with YOD each year combined with recruitment issues during the Covid-19 pandemic, when memory clinics were temporarily closed. The number of non-completers is in line with drop-out rates shown in previous longitudinal studies of dementia.^21,22^ As a result, the data did not fulfill the assumptions of parametric tests and we were restricted to non-parametric analyses. Given the relatively small sample size and multiple testing, the two statistically significant results should be interpreted with caution. A larger sample would have had more power to detect any differences over time or patterns, and we could have conducted repeated measures analyses, thereby reducing the risk of identifying significant findings by chance due to multiple testing.

The power of our analysis was further reduced by the amount of missing data, especially for the total score of QoL-AD. An early decision was made to not impute missing data as this increases the risk of biased estimates.^57^ Considering the diversity of scores and individual trajectories observed in the scatter plots, finding an appropriate method for imputing would probably have been difficult. The questionnaires were mostly sent to the participants by post for them to complete on their own, since this was preferred by most participants. In hindsight, conducting all ratings in an interview would probably have increased the response rate and decreased the amount of missing data; especially as we found the participants included in the qualitative interview study were more likely to be completers, even though there were also missing data in this group. This is important as it indicates the importance of personal contact to retain participants in a study. If a participant did not respond within a month, a new questionnaire was sent out together with a reminder. Sending reminders or personal contact, for example collecting questionnaire data in a telephone interview, have been described as having a positive effect on response rate.^58^ On the other hand, self-administration of questionnaires may increase the participants’ willingness to respond to sensitive questions.^59^

A strength of our study was the use of two instruments, the disease specific QoL-AD and the generic instrument RAND-36. Considering reliability, both instruments showing acceptable Cronbach's alpha values at baseline (all above 0.70). The decision to include QoL-AD was based on it being one of the most frequently used instruments to measure QoL in dementia,^12^ being sensitive to change,^35^ and valid and reliable when used in dementia,^11,34,35^ even without proxy report.^35^ In addition, we wanted to explore QoL and HRQoL from a broader perspective, with a generic instrument, RAND-36,^10,13^ which would make it possible to compare our participants to the general population. SF-36 (the commercial version of RAND-36) has been shown to have good correlation with the QoL-AD total score.^20^ SF-36 has also been described as reliable for persons with mild to moderate dementia^38^ with an MMSE score higher than 16 and having insight into their deficits.^20^

### Practice implication and future research directions

Despite the limitations mentioned above, we do believe that our findings can contribute to the field of knowledge. From a clinical perspective, they highlight the importance of a person-centered approach taking the individual's trajectory into account. In addition, the trend for persons with YOD to rate domains related to mental and social aspects of HRQoL lower than physical aspects indicates that support should target needs related to this. More longitudinal research, with larger samples, taking individual trajectories into account, is needed to better understand how QoL and HRQoL in YOD change over time, and if there are specific time points requiring extra attention. Further research is also needed to develop clinically relevant and sensitive instruments measuring QoL and HRQoL in relation to the specific needs of persons with YOD.

### Conclusion

Professionals and researchers meeting persons with YOD need to be aware of the individual variation in QoL and HRQoL trajectories, even if the scores seem to be stable over time at group level. In addition, since many individuals with YOD scored relatively high on the physical domain, and greater variation was observed on the social and mental domains, we should focus more on the mental and social aspects of HRQoL. Undoubtedly, there is a need for further longitudinal research on QoL and HRQoL in YOD, and the development of instruments that are sensitive and that target areas of relevance in these persons’ life situations.

## Supplemental Material

sj-docx-1-alz-10.1177_13872877251371305 - Supplemental material for Quality of life of persons with young-onset dementia: Repeated self-reported assessment over two years from diagnosis Supplemental material, sj-docx-1-alz-10.1177_13872877251371305 for Quality of life of persons with young-onset dementia: Repeated self-reported assessment over two years from diagnosis by Malin Aspö, Leonie NC Visser, Berit Seiger Cronfalk, Miia Kivipelto and Anne-Marie Boström in Journal of Alzheimer's Disease
