# Supplementary material for: Quality of life of persons with young-onset dementia: Repeated self-reported assessment over two years from diagnosis
Source: J Alzheimers Dis. 2025 Aug 29;107(4):1415–29. doi: 10.1177/13872877251371305 (PMC12495120; doi:10.1177/13872877251371305)
Supplement: sj-docx-1-alz-10.1177_13872877251371305 - Supplemental material for Quality of life of persons with young-onset dementia: Repeated self-reported assessment over two years from diagnosis [file sj-docx-1-alz-10.1177_13872877251371305.docx]

**Supplemental Material**

**Quality of life of persons with young-onset dementia: Repeated self-reported assessment over two years from diagnosis**

**Supplemental Figure 1a. RAND-36 Role limitations caused by physical health problems: total sample**

**Supplemental Figure 1b. RAND-36 Role limitations caused by physical health problems: completers**

**Supplemental Figure 1c. RAND-36 Role limitations caused by physical health problems: non- completers**


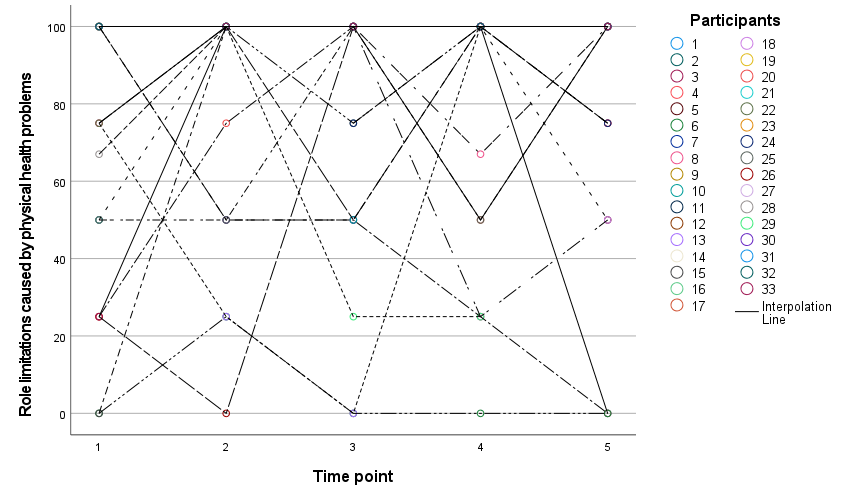

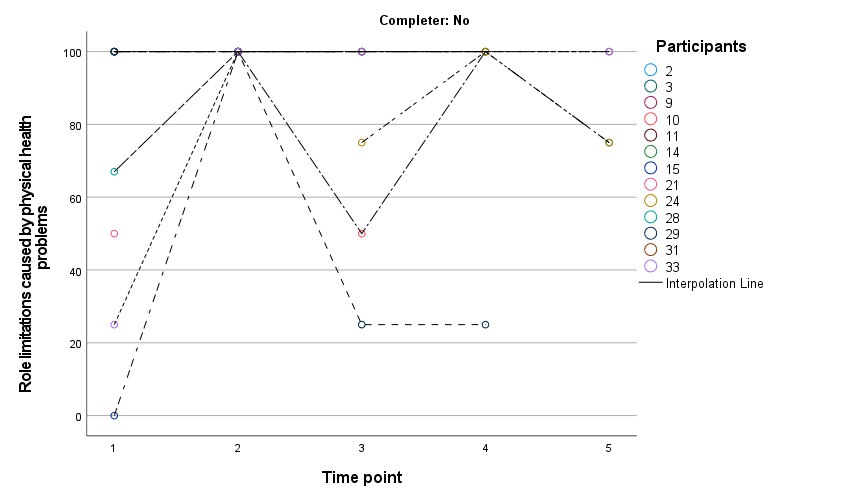

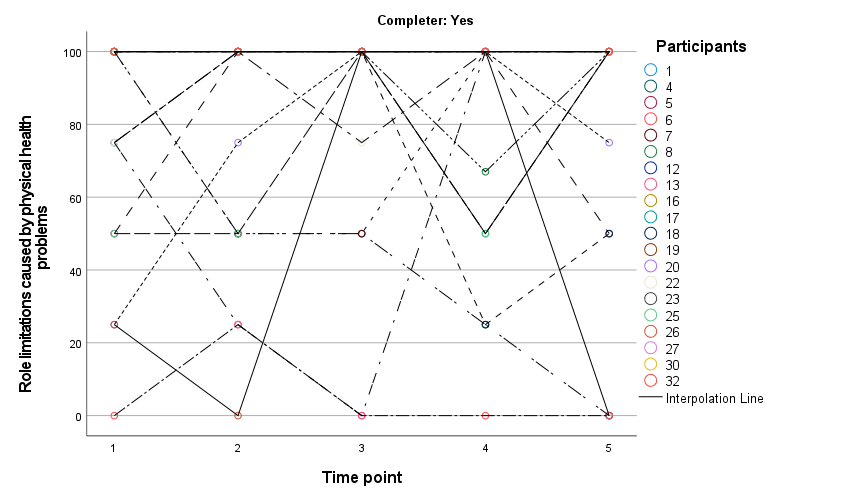

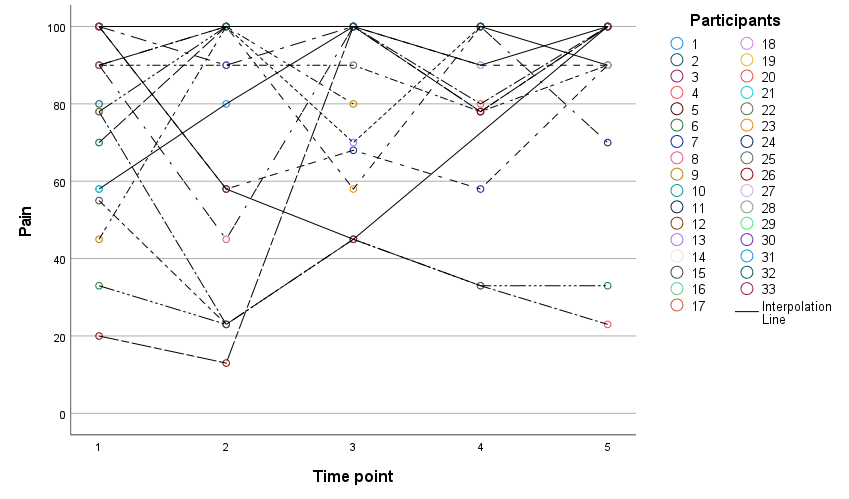

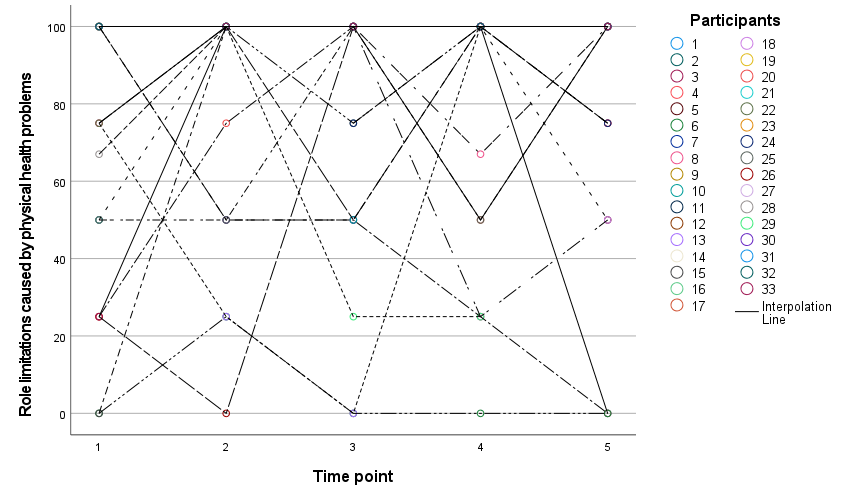

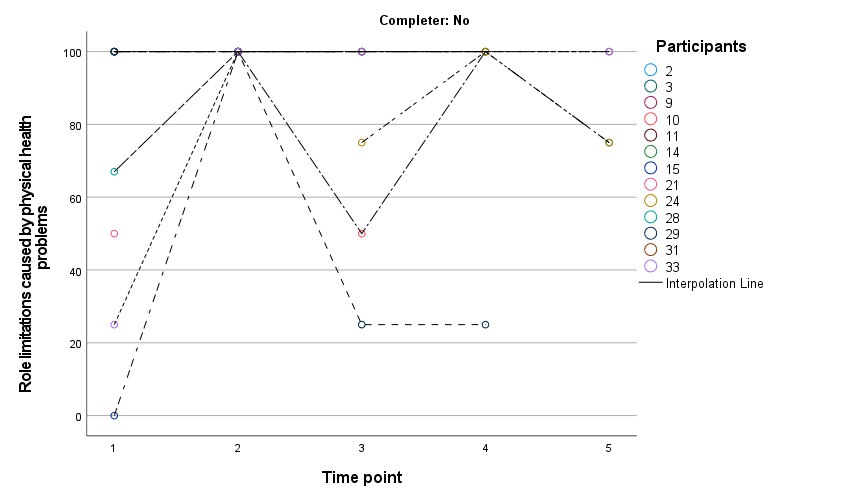

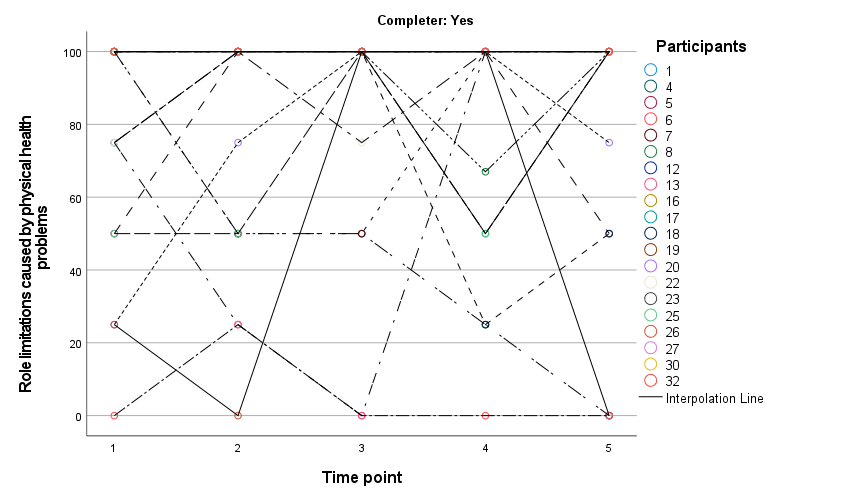

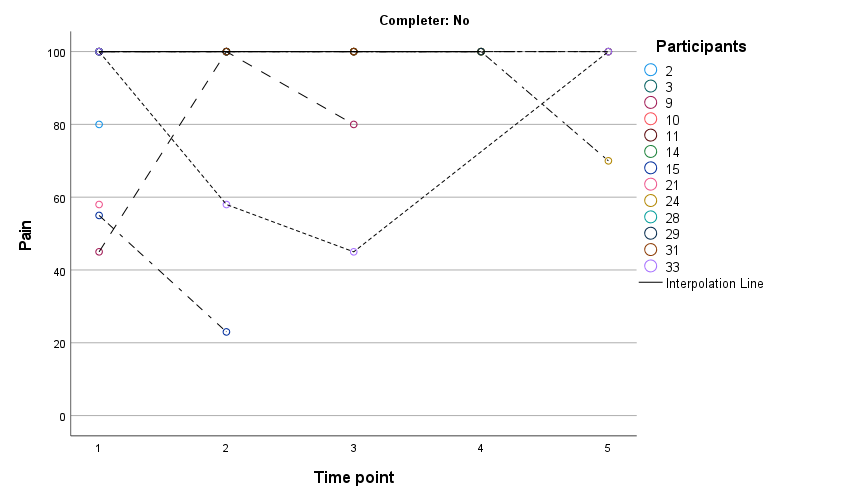

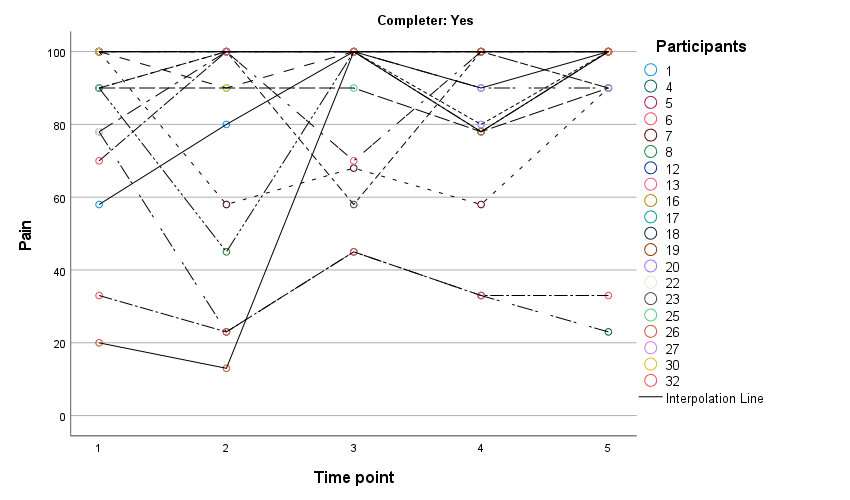


**Supplemental Figure 1.** RAND-36 Role limitations caused by physical health problems over time: individual trajectories plotted for the total sample (a), and for completers (b) and non-completers (c) separately. Scores ranging from 0 to 100. Higher scores indicate better HRQoL.

**Supplemental Figure 2a. RAND-36 Pain: total sample**

**Supplemental Figure 2b. RAND-36 Pain: completers**

**Supplemental Figure 2c. RAND-36 Pain: non- completers**

**Supplemental Figure 2.** RAND-36 Pain over time: individual trajectories plotted for the total sample (a), and for completers (b) and non-completers (c) separately. Scores ranging from 0 to 100. Higher scores indicate better HRQoL.


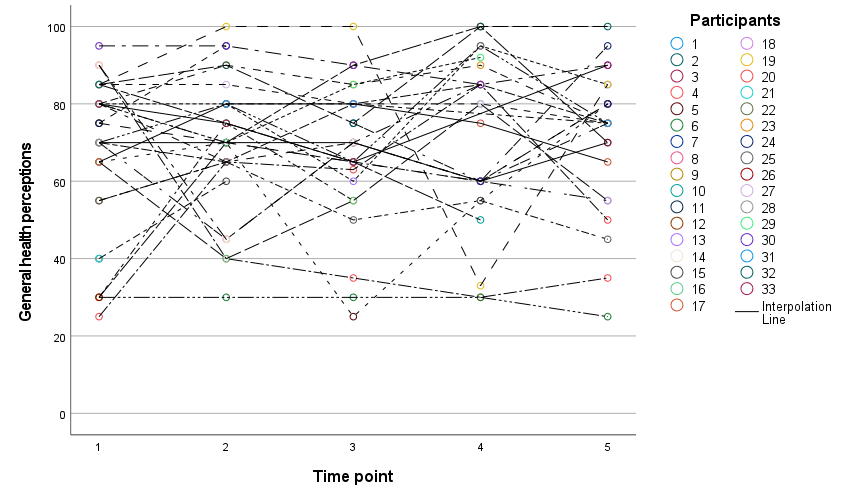

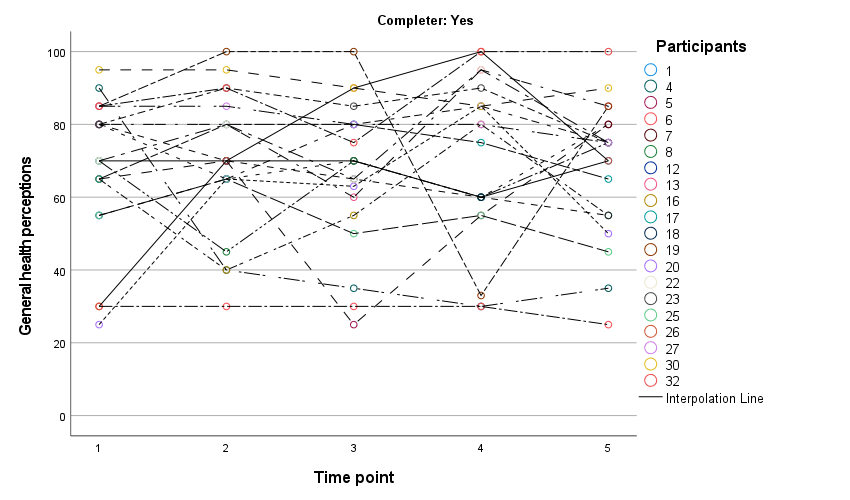

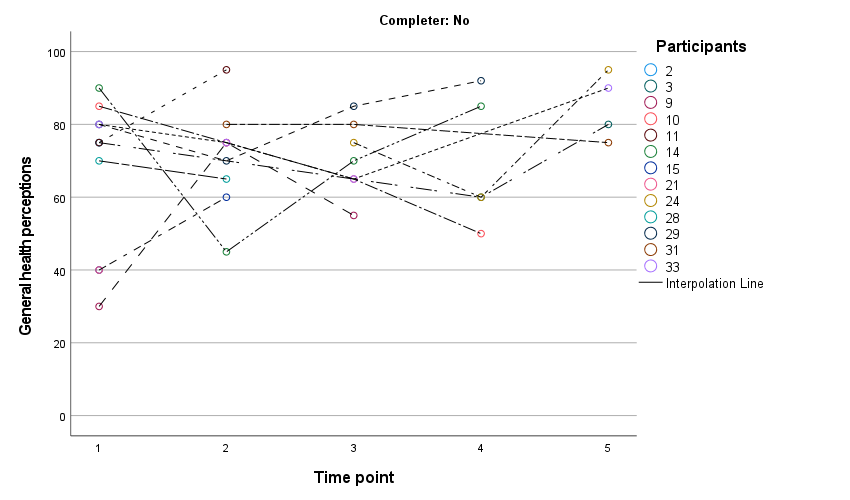


**Supplemental Figure 3.** RAND-36 General health perceptions over time: individual trajectories plotted for the total sample (a), and for completers (b) and non-completers (c) separately. Scores ranging from 0 to 100. Higher scores indicate better HRQoL.

**Supplemental Figure 3a. RAND-36 General health perceptions: total sample**

**Supplemental Figure 3b. RAND-36 General health**

**perceptions: completers**

**Supplemental Figure 3c. RAND-36 General health perceptions: non- completers**


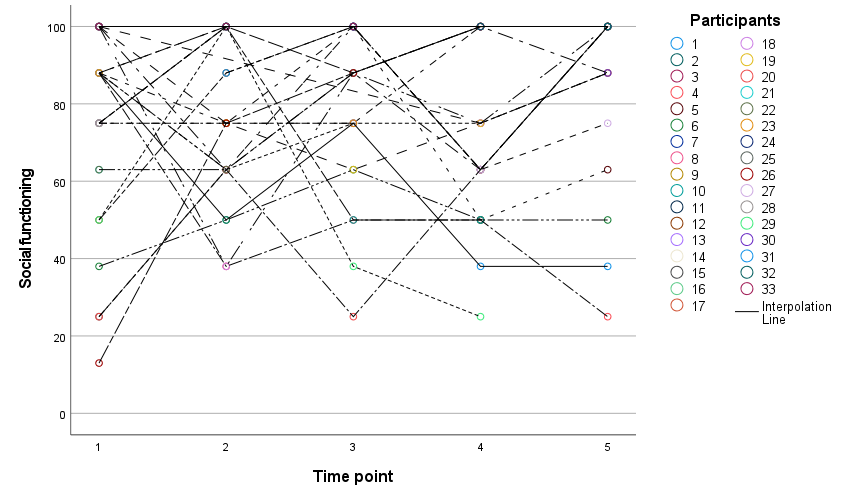

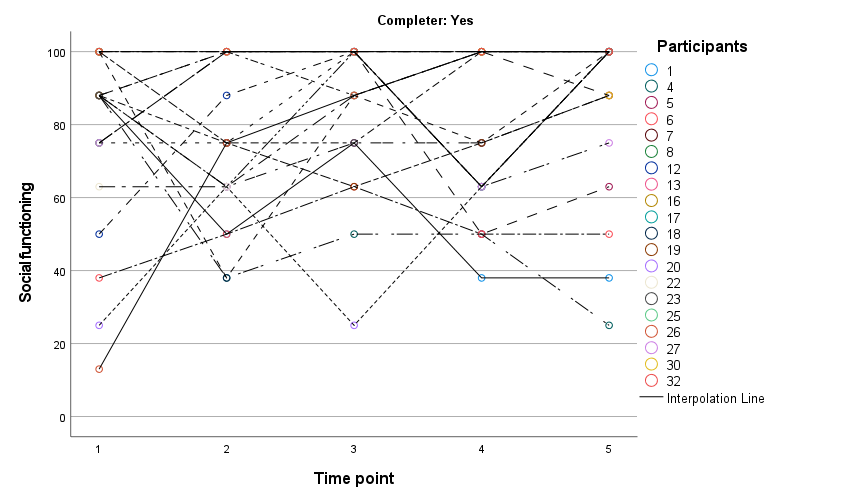

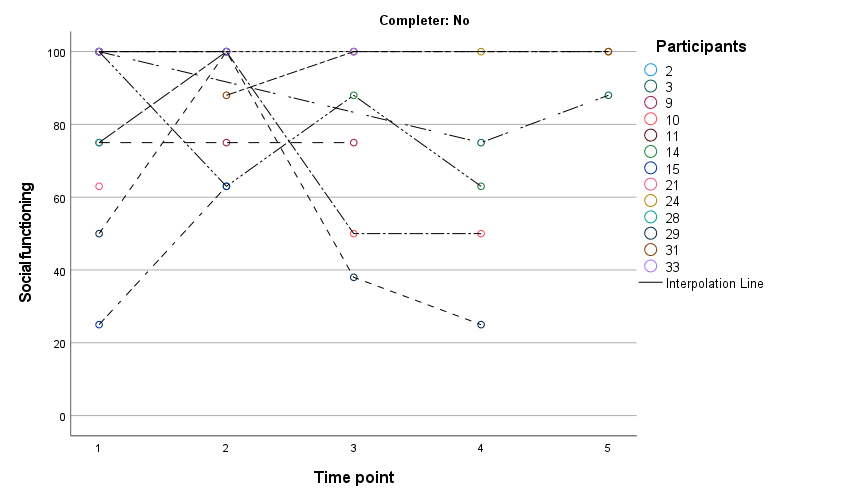


**Supplemental Figure 4.** RAND-36 Social functioning over time: individual trajectories plotted for the total sample (a), and for completers (b) and non-completers (c) separately. Scores ranging from 0 to 100. Higher scores indicate better HRQoL.

**Supplemental Figure 4a. RAND-36 Social functioning: total sample**

**Supplemental Figure 4b. RAND-36 Social functioning: completers**

**Supplemental Figure 4c. RAND-36 Social functioning: non- completers**


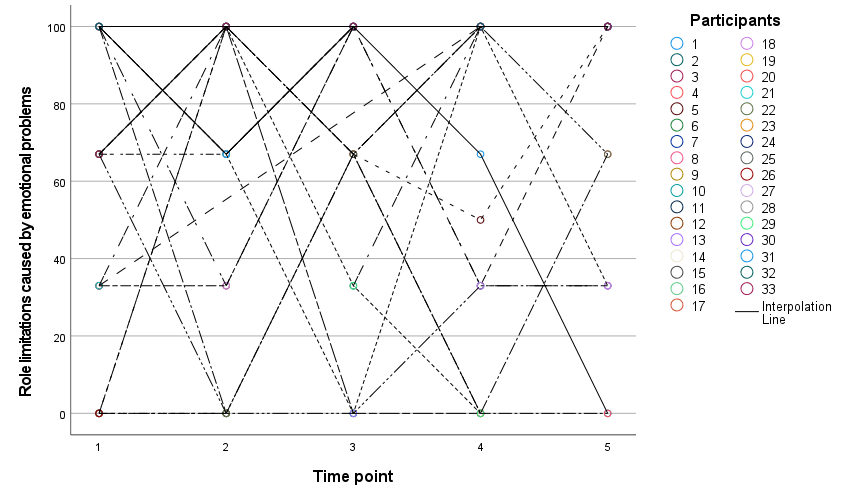

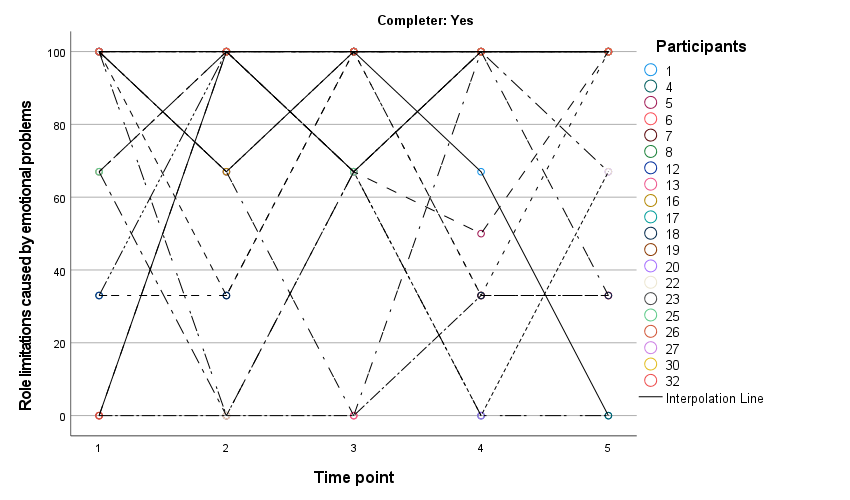

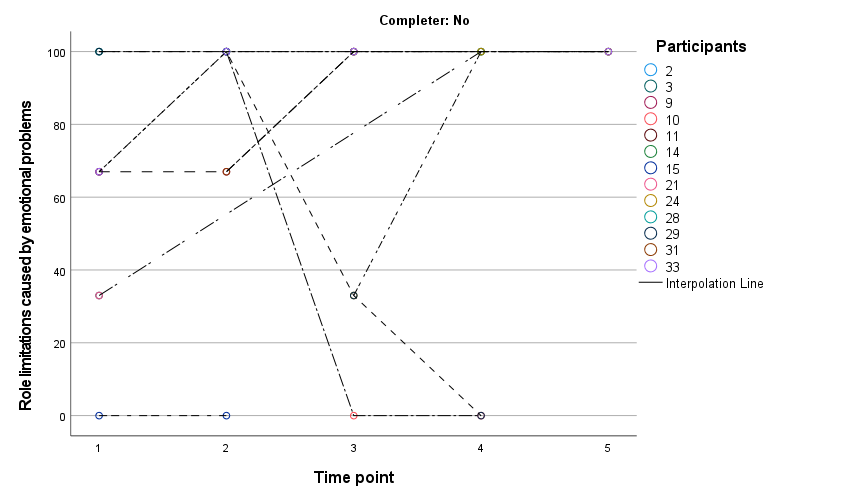


**Supplemental Figure 5a. RAND-36 Role limitations caused by emotional problems: total sample**

**Supplemental Figure 5b. RAND-36 Role limitations caused by emotional problems: completers**

**Supplemental Figure 5c. RAND-36 Role limitations caused by emotional problems: non- completers**

**Supplemental Figure 5.** RAND-36 Role limitations caused by emotional problems over time: individual trajectories plotted for the total sample (a), and for completers (b) and non-completers (c) separately. Scores ranging from 0 to 100. Higher scores indicate better HRQoL.


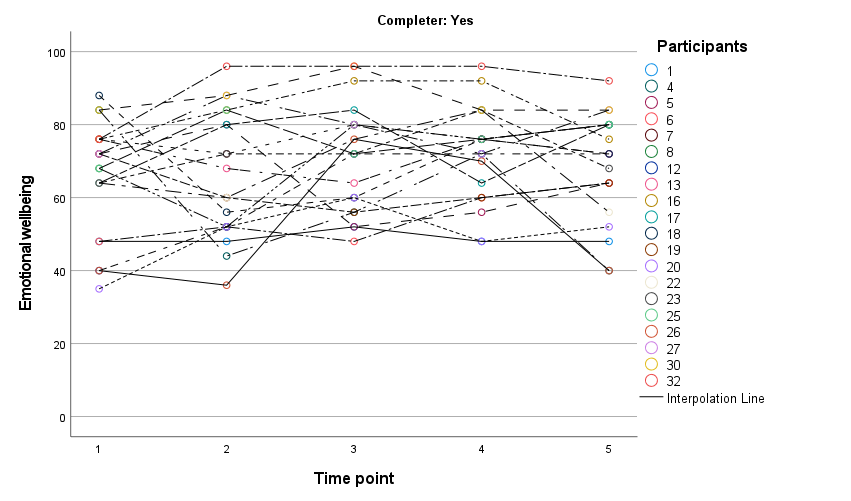

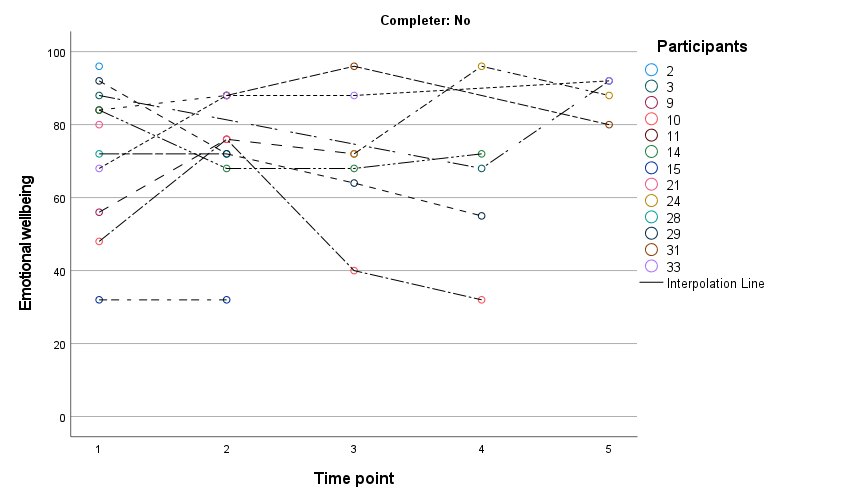

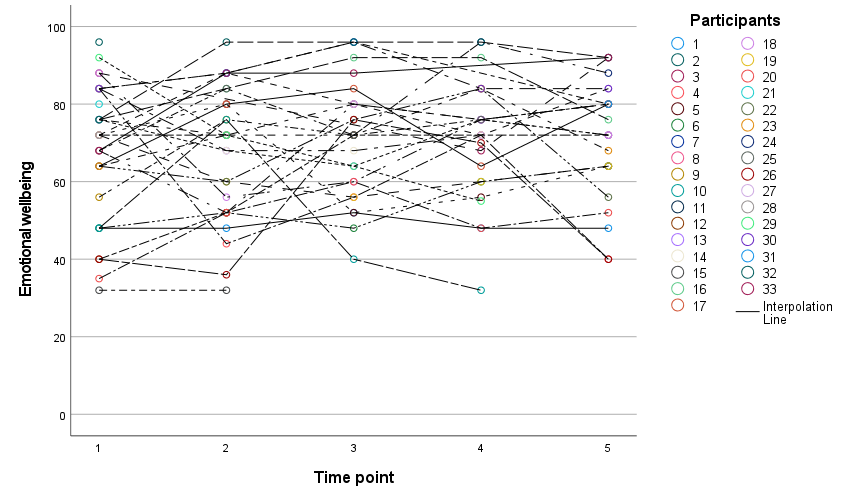


**Supplemental Figure 6.** RAND-36 Emotional wellbeing over time: individual trajectories plotted for the total sample (a), and for completers (b) and non-completers (c) separately. Scores ranging from 0 to 100. Higher scores indicate better HRQoL.

**Supplemental Figure 6a. RAND-36 Emotional wellbeing: total sample**

**Supplemental Figure 6b. RAND-36 Emotional wellbeing: completers**

**Supplemental Figure 6c. RAND-36 Emotional wellbeing: non- completers**
